# Extracellular Molecules Involved in Cancer Cell Invasion

**DOI:** 10.3390/cancers7010238

**Published:** 2015-01-26

**Authors:** Theodora Stivarou, Evangelia Patsavoudi

**Affiliations:** 1 Department of Biochemistry, Hellenic Pasteur Institute, Athens 11521, Greece; 2 Technological Educational Institute of Athens, Egaleo, Athens 12210, Greece

**Keywords:** cell invasion, metastasis, cell surface, extracellular

## Abstract

Nowadays it is perfectly clear that understanding and eradicating cancer cell invasion and metastasis represent the crucial, definitive points in cancer therapeutics. During the last two decades there has been a great interest in the understanding of the extracellular molecular mechanisms involved in cancer cell invasion. In this review, we highlight the findings concerning these processes, focusing in particular on extracellular molecules, including extracellular matrix proteins and their receptors, growth factors and their receptors, matrix metalloproteinases and extracellular chaperones. We report the molecular mechanisms underlying the important contribution of this pool of molecules to the complex, multi-step phenomenon of cancer cell invasion.

## 1. Introduction

Metastasis is one of the most important problems concerning mortality in cancer patients [1,2,3]. It is a multistep, complex process composed of a cascade of inter-connected events including: neo-vascularization, the escape of tumor cells from the primary tumor, a process known as local cancer cell invasion, migration through the extracellular matrix (ECM), intravasation, circulation and survival of the tumor cells in the blood or lymphatic circulation, extravasation and invasion of the tumor cells through the endothelium and basement membrane of the target site, and finally growth of the secondary tumor (colonization) [4,5]. During this multistage process, only very small numbers of cancer cells can survive, and give rise to secondary tumors. This type of cancer cell shows resistance under adverse conditions, such as tumor hypoxia and nutrient shortage [6], and chemo- and radio-therapies [7]. Moreover, these die-hard neoplastic cells must have the ability to self-renew and differentiate in order to create new tumor bulks at distant sites from the primary tumor [8]. Cancer Stem Cells (CSC) represent exactly the kind of tumor cells that possess all of these fundamental requisites for cancer cell invasion and metastasis [9].

Cancer cell invasion and secondary tumor outgrowth are regulated by numerous, interconnected molecular networks. In fact, there are many intracellular molecules belonging to the Wnt, Notch, Sonic Hedgehog, NF-κB, Ras/Raf/MEK/MAPK, as well as the AKT/ERK signaling pathways, which control every aspect of each of the stages of cancer cell invasion [4,5,10]. On the other hand, extracellular molecules also contribute in a critical way to the progress of cancer cell invasion. These molecules can be (a) part of the ECM, (b) secreted in the ECM (c) secreted but also attached on the cell surface (d) cell membrane proteins such as receptors. In this review, we focus on the function and regulation of extracellular molecules that take part in cancer cell invasion and metastatic processes (Table 1).

**Table 1:** Extracellular molecules involved in cancer cell invasion.

Category	Molecule Name	Molecule Type	Molecules Co-involved in Cancer Cell Invasion
**ECM MOLECULES**	Hyaluronan (HA)	glycosaminoglycan	CD44
	Fibronectin (FN)	glycoprotein	eHSP90, HSP90, MMP-9, MMP-9, FAK/PI3K/AKT/ERK/NF-κB, PEDF
	SIBLING	Small Integrin-Binding Ligand, N-linked Glycoprotein	Pro-MMPs, MMP-2, MMP-9, MMP-3, αvβ3 integrin, FAK/MEK/ERK/NF-Κβ pathway, CD44v6
**ECM RECEPTORS**	Integrins	Cell surface receptors	Fibronectin, MMP-9, MMP-2, FAK/ILK/, ERK/, PI3K/NF-κB signaling cascades EGFR, osteopontin
	CD44	Cell surface receptors	Hyaluronan (HA), osteopontin
**GROWTH FACTORS**	TGF-β	Growth factors	TBRI, TBRII, Erk, Ras
	Heregulin	EGF-like growth and differentiation factor	ErbB3, ErbB4, PAK-1, AMF
GROWTH FACTOR RECEPTORS	EGFR	Cell surface receptor	TGF-α, Grb2, Ras/Raf/MEK/MAPK
	HER-2	Cell surface co-receptor	HER-3, eHSP90, MAPK, PI3K/AKT
	IGF-R	Cell surface receptor	IGFs, IRS-2, PI3K/AKT, Ras/Raf/MAPK
MATRIXMETALLO-PROTEINASES	Matrix Metalloproteinase (MMP)-9	Zinc endopeptidase	eHSP90, HSP90, Rab40b, VAMP-4, gelatin type IV collagen, VEGF, bFGF
	Matrix Metalloproteinase (MMP)-2	Zinc endopeptidase	gelatine, type IV collagen, eHSP90, HSP90, Rab40b, VAMP-4, VEGF, bFGF
	CD10	Zinc-dependent metalloproteinase	Twist1
CHAPERONES	eHSP90	Chaperone	Cdc37, FN, HER-2, EGFR, pro-MMP-2, pro-MMP-9
	eCdc37	Co-chaperone	HSP90, eHSP90, HER2, EGFR, Raf1, CDK4, EGFRvIII, Peuth-Jeghers cancer syndrome-associated kinase
LRP-1	LRP-1	Low-density lipoprotein (LDL) receptor	Nexin-1 (PN-1), Erk pathway, MMP-9, eHSP90, EphA2, AKT1, AKT2

## 2. ECM Molecules

### 2.1. Hyaluronan

**Figure 1:**
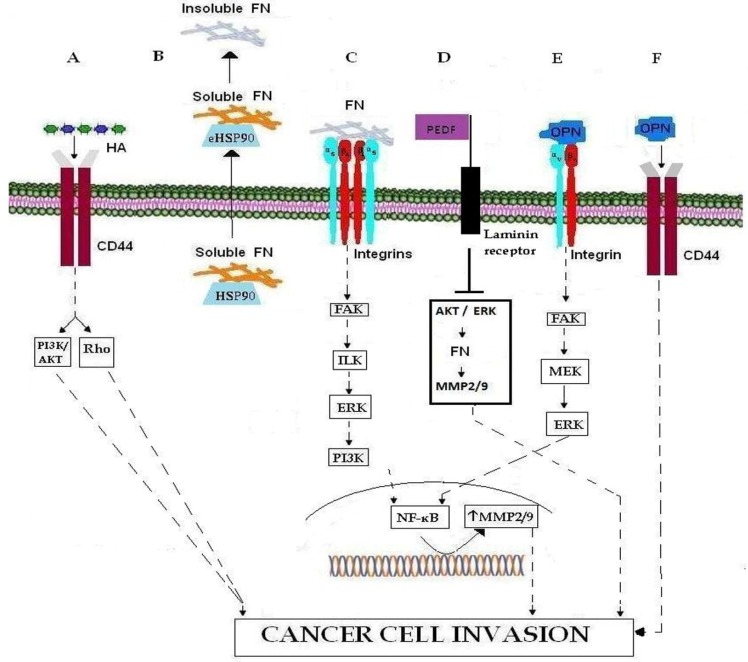
Important ECM molecules and ECM receptors involved in cancer cell invasion. (**A**) Binding of HA to CD44 promotes cell invasion. HA-CD44 interaction promotes invasion via the PI3Κ/AKT and Rho signaling pathways. (**B**) The transport of FN, in its soluble form, to the membrane occurs through the chaperone activity of intracellular HSP90, while eHSP90 regulates the conversion of soluble FN to its insoluble ECM form. (**C**) Binding of FN to integrin results in MMP-2 and MMP-9 over-expression via the FAK/ILK/ERK/PI3K/NF-κB pathways, and thereby leading to ECM degradation and cancer cell invasion. (**D**) PEDF binding to integrin results in MMP-2 and MMP-9 down-regulation through inhibition of AKT/ERK signaling pathway. (**E**) OPN binding to integrin promotes cancer cell invasion by inducing MMP-9 over-expression through the FAK/MEK/ERK/NF-κB pathway. (**F**) Binding of OPN to CD44 promotes cell invasion.

Hyaluronan (HA), also known as hyaluronic acid, constitutes the major glycosaminoglycan present in the ECM. HA binds mainly to CD44 receptor (Figure 1A) and promotes tumor growth, survival as well as cancer cell invasion [11]. Moreover, data suggest that HA forms a protecting covering for cancer cells against cytotoxic and chemotherapeutic agents, and that augmented HA synthesis leads to a less dense matrix that facilitates cancer cell motility and invasion. Regarding tumor progression, it has been shown that HA promotes tumor-associated angiogenesis [11,12] and that the expression of this molecule and hyaluronan synthase (HAS), as well as the rate of HA synthesis, are increased in highly metastatic breast carcinoma cells [13,14].

### 2.2. Fibronectin

ECM glycoprotein fibronectin (FN), a major cell-matrix and cell-cell adhesion mediator, is involved in the regulation of embryogenesis, mesoderm formation, tissue repair, cell migration, differentiation, cell growth as well as certain pathological disorders such as fibrosis, atherosclerosis, tumor invasion and metastasis [15,16,17,18]. FN is secreted by cells as a soluble dimer that is then assembled into an insoluble network of fibers. The change of FN conformation from soluble to insoluble form begins after its secretion, when it binds to cell surface integrins and exposes self-association sites which allow the creation of an insoluble form [19,20,21]. Regarding the regulation of FN function, Hunter *et al.* suggest that intracellular HSP90 acts as a chaperone for the stabilization and/or transport of soluble FN followed by its export from the cell (Figure 1B). Once secreted, extracellular HSP90 (eHSP90) promotes the conversion of soluble FN to its insoluble form (Figure 1B). The intra- and extracellular roles of HSP90 as an FN chaperone can be considered as another HSP90 mechanism, which promotes cell migration and metastasis through the degradation and remodeling of ECM [17]. FN over-expression has been reported in specimens of various tumor types such as breast, lung, thyroid and esophageal cancer [22]. Additionally, FN over-expression has been correlated with poor clinical outcome in breast cancer patients, whilst its involvement in breast cancer invasion and metastasis has been demonstrated to involve the up-regulation of matrix metalloproteinases (MMPs) MMP-2 and MMP-9 via the FAK/ILK//ERK/PI3K/NF-κB cascade of pathways [23,24] (Figure 1C). Most recently Hong *et al.* [16] proposed a new model regarding the down-regulation of FN by pigment epithelium-derived factor (PEDF), a molecule well-known for its important anti-cancer role, mainly through the inhibition of angiogenesis and the induction of tumor differentiation and apoptosis in various types of cancer. In particular, they showed that PEDF inhibits MMP-2 and MMP-9 expression by binding to the laminin receptor and consequently inhibiting the AKT/ERK tumorigenic pathway, thereby down-regulating FN expression (Figure 1D).

### 2.3. SIBLING

The Small Integrin-Binding Ligand, N-linked Glycoprotein (SIBLING) family includes bone sialoprotein (BSP), osteopontin (OPN), dentin matrix protein 1 (DMP1), dentin sialoprotein (DSPP), and matrix extracellular phosphoglycoprotein (MEPE). They comprise a class of non-structural ECM proteins. Expression of SIBLING family members was first characterized in mineralized tissue including bone and teeth. Additionally, SIBLING proteins are localized in neoplastic tissues and induce metastasis [25,26]. Elevated SIBLING expression has been associated with an analogous increased expression of MMPs in breast, stomach, colon, ovarian, rectal and lung cancers [25]. Amongst the SIBLING proteins mentioned above, OPN is a secreted phosphoprotein characterized as a biomarker of tumor metastasis because increased OPN expression was found within tumor cells and in the surrounding stroma of multiple human cancers [27,28,29,30,31,32,33,34]. Nowadays, OPN is considered a serum biomarker in predicting tumor metastasis. Elevated OPN levels can be specific in predicting disease progression in head and neck, gastric, renal, hepatocellular, lung, and pancreatic cancers as well as uveal melanoma. Additionally, it has been established that OPN is a strong prognostic indicator for overall survival as its circulating levels are proportional with tumor stage and metastasis [35,36]. Two main mediators of OPN signaling pathways are ανβ integrins and CD44. OPN binds various types of integrins, such as ανβ3 which participates in the metastatic phenomenon in several ways. ανβ3-OPN interaction promotes cancer cell migration and invasion in prostate and breast cancer as well as in chondrosarcoma where OPN-ανβ3 binding leads to MMP-9 up-regulation through the FAK/MEK/ERK/NF-Κβ pathway [37,38,39,40] (Figure 1E). Moreover, OPN-ανβ3 integrin ligation promotes neo-vascularization by up-regulating endothelial cell migration, survival and lumen formation during angiogenesis [41,42,43,44,45]. Finally, OPN interaction with CD44v6 is observed in metastasis of breast, hepatocellular, pancreatic, lung, colorectal cancers and lymphomas [26,46,47,48,49,50,51] (Figure 1F).

Primary tumor formation and metastatic processes are clearly the result of the co-participation of genetically modified tumor and normal cells. OPN is mainly secreted by tumor cells while in myeloid cells OPN is localized intracellularly. Most recently, Sangaletti *et al.* clarified an aspect of the dual role of OPN whereby tumor cells secrete OPN in order to support their survival in the blood circulation, whereas both tumor- and host-derived OPN, particularly from myeloid cells, render the metastatic site more immunosuppressive [52].

## 3. ECM Receptors

### 3.1. Integrins

Integrins are the major and most characterized cell surface receptors of several ECM proteins such as laminin, fibronectin, collagen IV and vitronectin. Integrins are composed of non-covalent, heterodimeric complexes of an α and β subunit [53]. Many members of the integrin family, such as α5β1, α8β1, αIIbβ3, αVβ3, αVβ5, αVβ6, and αVβ8 recognize an Arg-Gly-Asp (RGD) motif within their ligands, which include FN, fibrinogen, vitronectin, von Willebrand factor, and various other large glycoproteins [54]. Both the α and β subunits are transmembrane glycoproteins. As the cytoplasmic tails of integrins are devoid of enzymatic features, they transduce signals by associating with adaptor proteins that connect the integrin to the cytoskeleton, cytoplasmic kinases, and transmembrane growth factor receptors [55]. Integrins constitute the mediators between ECM and the actin cytoskeleton with focal adhesion sites representing the regions of signal transduction controlling proliferation, differentiation, survival, wound healing, migration, tumorigenesis, *etc.* [56]. It has been suggested that bone metastasis derived from advanced prostate cancer process is characterized by the integrin-mediated interaction of metastatic cancer cells and bone microenvironment [57]. In fact, it has been shown that in the majority of tumors, ανβ3 integrin is the prime initial receptor to support adhesion and migration to bone matrix. The crucial role of integrins in cancer cell invasion is additionally evidenced by an α5β1 integrin-FN interaction (Figure 1C), which accelerates cell invasion of SiHa cervical cancer cells and promotes the expression and activation of pro-MMP-9, as well as moderate change of pro-MMP-2 activity through the FAK, ILK, ERK, PI3K and NF-κB signaling cascade [58]. Moreover, an α5β1 integrin-FN interaction was found to up-regulate MMP-9 expression and activity in the highly metastatic MDA-MB-231cancer cell line. Additionally, the expression of ανβ3 integrin has been found significantly higher in pancreatic primary tumors with lymph node infiltration, as compared to those without node metastasis, while tumors with high ανβ3 integrin expression showed significantly higher MMP-2 activation ratios than did tumors with low expression of this receptor [59]. Regarding breast cancer-bone metastasis, Takayama *et al.* reported that breast cancer cells which express αvβ3 integrin acquire the ability to adhere to bone matrix in breast cancer bone metastasis [60]. Finally, a crosstalk between ανβ3 integrin and epidermal growth factor receptor (EGFR) has been shown, through which cancer cell invasion and metastasis are stimulated [61].

### 3.2. CD44

The CD44 receptors are cell-surface molecules which mediate cell-matrix and cell-cell interactions [26,47]. They constitute a family of transmembrane glycoproteins encoded by a single gene. Alternative splicing and variation in N- and O-glycosylation give rise to the various CD44 isoforms distinguishable by their differential roles in breast cancer CD44s, one of the most expressed CD44 isoforms, is up-regulated in primary tumors but correlates with overall patient survival [62,63]. In fact, it has been shown that CD44s inhibits cancer cell invasion since loss of CD44s *in vivo* resulted in a marked promotion of cancer metastasis to the lung in a metastatic mouse model of breast carcinoma whereas tumor onset and tumor size were unaffected [63,64]. On the other hand, Rys *et al.* revealed a strong correlation of CD44ν3 isoform with tumor infiltration by T lymphocytes and cancer metastasis to draining lymph nodes combined with a loss of p53 protein expression [13,64].

Furthermore, as mentioned above, CD44 represents the main receptor of HA [13,65] whose binding with CD44 promotes signaling pathways that induce tumor growth, survival as well as cancer cell invasion [11]. In particular, binding of HA with CD44ν3 triggers downstream Rho and PI3K-AKT signalling pathways, inducing breast cancer cell growth, and invasion (Figure 1A).

Nowadays, CD44 is an established marker of cancer stem populations in breast, prostate, pancreas, ovarian and colorectal cancers [66,67,68]. In particular, the CD44^+^/CD24^low/−^ combination represents the molecular phenotype of breast CSC sub-population which represents the part of the tumor that can survive during colonization and promote cancer cell invasion. In fact, breast CSC constitute the only cell sub-population of this type of neoplasia that can effectively induce the creation of tumors when injected to immunosuppresed mice [69].

## 4. Growth Factors

### 4.1. TGF-β

**Figure 2:**
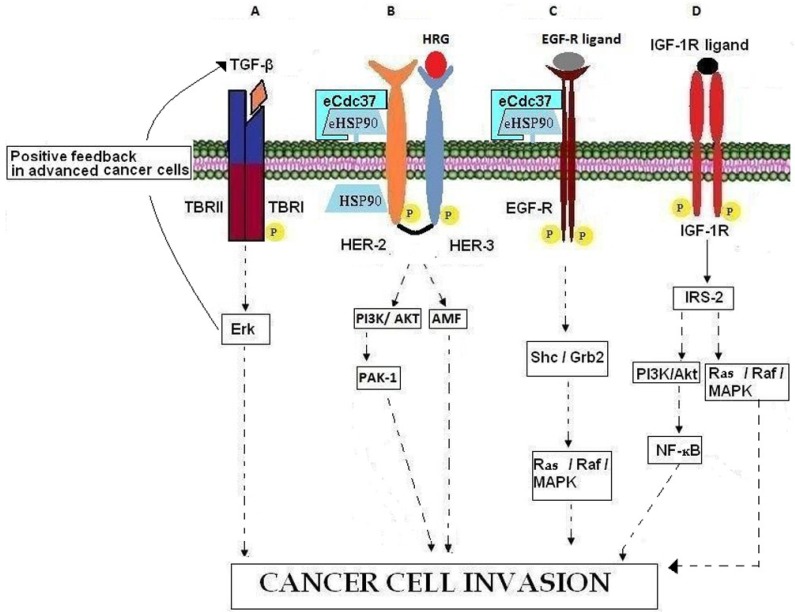
Involvement of growth factors, growth factor receptors, and chaperones in cancer cell invasion. (**A**) In advanced cancer cells, TBRII activation promotes cancer cell invasion by binding over-expressed TGF-β and activating Erk which induces a positive feedback loop by further increasing TGF-β. (**B**) HRG binding to HER-3 leads to the formation of HER-3/HER-2 heterodimers, the activation of downstream kinase signaling pathways and in actin re-arrangement and cell invasion. HSP90 contributes to intracellular HER-2 stabilization while eHSP90-eCdc37-HER-2 heterocomplexes are necessary for HER-2 heterodimerization with HER-3, leading to cell invasion through PI3K/AKT and AMF signaling pathways. (**C**) EGFR binding to its ligands leads to activation of the Ras/Raf//MAPK pathways resulting in cancer cell invasion. The chaperoning activity of eHSP90, associated with co‑chaperone eCdc37, is necessary for EGFR stabilization. (**D**) Binding of IGF-1R with its ligand results in IGF-1R auto-phosphorylation, recruitment of adaptor proteins such as IRS-2 and subsequent activation of PI3K/AKT and Ras/Raf/MAPK pathways which promote the invasion processes.

Transforming growth factor β (TGF-β) belongs to a large family of polypeptide growth factors that includes activins, inhibins, and bone morphogenetic proteins (BMPs). There are three known mammalian TGF-β isoforms (TGF-β1, TGF-β2, TGF-β3), which are closely related both structurally and functionally. These isoforms are secreted as latent precursor molecules that are activated by proteolytic cleavage, interaction with integrins, or pH changes in the local environment [70,71,72]. Active TGF-β is implicated in many regulatory activities that influence development, tissue repair, immune defense, inflammation and tumorigenesis [70,73]. The biological effects of TGF-β are mediated through specific receptors (TBRI and TBRII), which are transmembrane serine/threonine kinases (Figure 2A). TGF-β is involved in tumor cell invasion by participating in epithelial mesenchymal transition (EMT) [74,75,76], by enhancing angiogenesis [77] and by mediating immune evasion of tumor cells [78,79]. TGF-β is known to inhibit the cell cycle in benign cells and early stage cancer cells while at the same time it promotes progression of the cell cycle and metastasis in advanced cancer cells [78,79,80,81]. This phenomenon is known as the TGF-β paradox [82]. Recently, Zhang *et al.* [83] reported that differential activation of Erk in cancer cells is the underlying molecular mechanism of the TGF-β paradox (Figure 2A). More precisely, the inhibition or progression of cell cycle is due to inactivation or activation of the cell proliferation regulator Erk, respectively [83,84].

### 4.2. Heregulin

Heregulin (HRG; also called neregulin (NRG), Neu differentiation factor (NDF), glial growth factor (GGF), and acetylcholine receptor-inducing activity (ARIA)) is a member of the EGF-like growth and differentiation factors and binds with high affinity to the receptors ErbB3 and ErbB4 [85]. The HRG gene family consists of four members, HRG-1, HRG-2, HRG-3*,* and HRG-4 [86,87,88,89,90] of which a multitude of different isoforms are synthesized by alternative exon splicing [91], showing various tissue distributions, variable potencies, different receptor specificities, and variable biological functions. HRG has been implicated in developmental processes [85,92,93,94], as well as in the patho-physiological processes of psychiatric diseases, cardiac diseases, and various types of cancer. Although increasing data indicate that HRG-2, HRG-3, and HRG-4 may play a role in malignancy, most research interests have focused on the HRG-1 gene. HRG-1 is over-expressed in 30% of human breast cancer patients [95]. On the other hand Raj *et al.* have reported that low levels of HRG1 in cases of locally advanced breast cancer, are associated with poor prognosis [96]. HRG participates in tumor growth through induction of angiogenesis and invasion. In particular it has been shown that HRG induces EMT process and cell migration in SK-BR-3 and MCF7 breast cancer cells by binding to the HER-3 receptor and signaling through the PI3K/AKT pathway [85,92,93,94,97,98] (Figure 2B). HRG induced cell migration through PI3K takes place also by regulating PAK-1 and enhancing the formation of lamellipodia, membrane ruffles, stress fibers and fidopodia [99]. HRG-induced progression of breast cancer cells into a more aggressive phenotype also involves the regulation of Autocrine Motility Factor (AMF). In fact, it has been shown that HRG stimulates cell motility-associated changes such as cell scattering and actin re-organization by up-regulating the expression of AMF [100]. HRG stimulates the up-regulation of proteins implicated in cell invasion acting both as a ligand which binds its cell surface receptor(s) and activates downstream signaling pathways and as an intracellular mediator of the expression of invasion-related genes. In fact, reported data suggest that HRG may regulate transcription indirectly by recruiting co-factors considered essential for transcriptional control [101,102].

## 5. Growth Factor Receptors

### 5.1. ErbB Receptors

The ErbB family of receptor tyrosine kinases (RTK) includes four distinct receptors: the EGFR (also known as ErbB-1/HER-1), ErbB-2 (neu, HER-2), ErbB-3 (HER-3) and ErB-4 (HER-4) [103,104]. With respect to ErbB-receptor binding, ErbB ligands can be classified into three groups: (a) those that bind specifically to EGFR, in particular, transforming growth factor α (TGFα) and amphiregulin (AR); (b) those that show dual specificity by binding EGFR and ErbB-4, specifically betacellulin (BTC), heparin-binding growth factor (HB-EGF) and epiregulin (EPR); (c) the neuregulins (NRGs) which can be divided in two sub-groups based upon their capacity to bind HER-3 and HER-4 (NRG-1 and NGR-2) or only HER-4 (NRG-3 and NGR-4) [86,87,89,105,106]. None of the EGF family of ligands bind HER-2. Ligands binding to the extracellular domain of the respective receptors induce homo- or hetero-dimerization of ErbBs. Dimerization consequently stimulates intrinsic tyrosine kinase activity of the receptors and triggers auto-phosphorylation of specific tyrosine residues within the cytoplasmic regulatory domain. These phosphorylated tyrosines serve as binding sites for various adaptor proteins such as Shc, Grb7, Grb2, Crk, Nck, the phospholipase Cγ (PLCγ), the intracellular kinases Src and PI3K, the protein tyrosine phosphatases SHP1 and SHP2 and Cbl E3 ubiquitin ligase [107,108]. EGFR ligands and receptors induce activation of the Ras/Raf/MEK/MAPK pathway through either Grb2 or Shc adaptor proteins [109,110,111]. These signaling events eventually result in cell proliferation, angiogenesis, resistance to apoptosis, migration and metastasis (Figure 2C). In fact, in normal tissues the availability of EGFR ligands is regulated in order to ensure that the kinetics of cell proliferation precisely match the tissues’ requirements for homeostasis. In the case of neoplasia however, the EGFR is often chronically stimulated, either by EGFR ligands that are over-produced within the tumor microenvironment [106,112] or as a result of EGFR mutation that causes spontaneous receptor activation [113]. EGFR is expressed in various types of neoplasia including those in the lung, head and neck, colon, pancreas, breast, ovary, bladder and kidney as well as in gliomas [114]. The over-expression of EGFR and TGFα by neoplasias confers a more aggressive phenotype by inducing cancer metastasis, resistance to chemotherapy and poor prognosis [115,116,117]. Given its massive presence in several tumors and its key role in metastasis, EGFR is defined as a principal target in anti-cancer therapies [118].

HER-2 is considered a ligandless receptor which shows preferred heterodimerization with HER-1, HER-3, and HER-4 [104,110,119]. HER-2 functions as a co-receptor to mediate signal transduction resulting in cell motility, mitogenesis, apoptosis, angiogenesis and/or cell differentiation. Any alteration of the tightly regulated HER-2 receptor signaling pathways results in major cellular abnormalities and tumorigenesis. HER-2 over-expression is strongly associated with increased progression and metastasis in human breast and prostate cancer [104,110,119,120,121]. Whilst it had been shown already that intracellular HSP90 contributes to the stability of HER-2 via its cytoplasmic kinase domain [122,123,124]. Sidera *et al.* [125] reported in 2008 that the molecular interaction of cell surface HSP90 with the extracellular domain (ECD) of HER-2 is necessary for breast cancer cell invasion. More specifically, they showed that this interaction is essential for receptor activation and subsequent heterodimerization with HER-3 which in turn mediates signal transduction pathways via MAP kinase and PI3K/AKT, leading to actin re-arrangement necessary for cell motility (Figure 2B). It has been reported previously that the ECD of HER-2 constitutively adopts an extended configuration with its dimerization arm exposed, suggesting that it is always poised to form heterodimers with ligand-activated forms of ErbB-receptors [110,126,127]. Taking this into consideration Sidera *et al.* speculated that surface HSP90 interacts with the HER-2 ectodomain in order for the receptor to maintain its active conformation. Finally, it should be noted that HER-2–HER-3 heterodimerization is essential for mediating the effects of growth factors such as HRG on cell motility. HRG binds to HER-3 to activate downstream kinase signaling pathways which lead to actin re-arrangement and cell invasion [125].

### 5.2. IGF-R

The insulin-like growth factor (IGF) machinery comprises (a) the circulating ligands; insulin-like growth factor-1 (IGF-1), IGF-2 and insulin, (b) multiple receptors; IGF-1R, insulin receptor (IR), hybrid receptors containing one chain of IGF-1R and one chain of IR (IGF-1R/IR-A, IGF-1/IR-B) and IGF-2R, (c) multiple adaptor proteins. Under physiological conditions, IGF-1R activation is implicated in fetal growth as well as in linear growth of the skeleton and other organs [128]. In the case of neoplasia, IGF-1R is frequently over-expressed inducing proliferation, cancer cell motility and adhesion, as well as inhibition of apoptosis. It is known to promote metastasis in various cancers, including those of the colon, pancreas, prostate and breast [129,130,131]. IGF-1R is activated by IGFs present in the extracellular environment, in an endocrine, paracrine or autocrine manner. Upon ligand binding, IGF-1R becomes auto-phosphorylated and subsequently recruits specific docking intermediates, including insulin-receptor substrate-2 (IRS-2), that activate PI3K/AKT and Ras/Raf/MAPK pathways in order to promote cell motility and pro-metastatic behaviour in breast cancer cells [129,132,133] (Figure 2D). In models of breast cancer bone metastasis, IGF-1R activation promotes motility of bone-metastatic cells [134]. In this context, it has been reported that bone-derived IGFs, which are released from bone in substantial amounts by osteoclastic bone resorption, activate IGF-IR/AKT/NF-kB signaling pathways in breast cancer cells that are colonizing the bone, thereby increasing their proliferation, decreasing apoptosis and thus promoting the development and progression of bone metastases [135,136].

## 6. Matrix Metalloproteinases

### 6.1. MMPs

**Figure 3:**
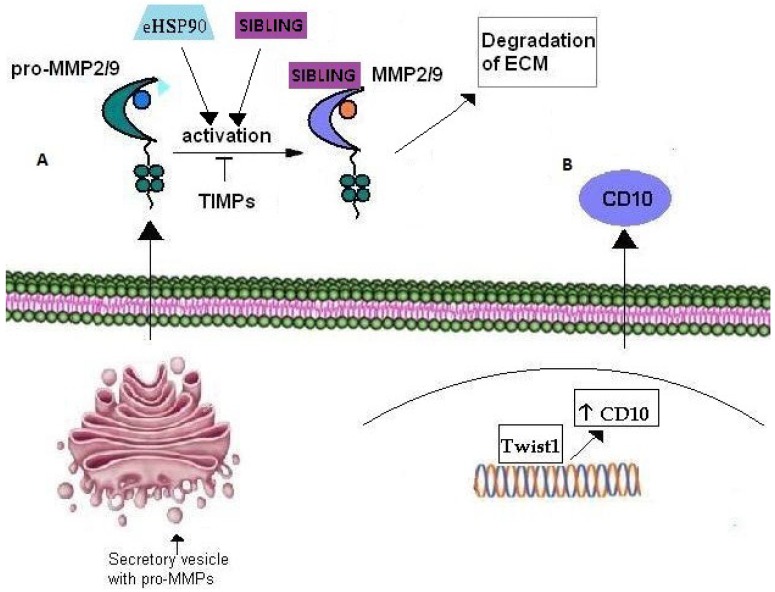
Role of Matrix Metalloproteinases in cancer cell invasion. (**A**) Pro-MMP2/9 are transported through secretory vesicles of the Golgi network to the plasma membrane. Their activation occurs extracellularly through their interaction with eHSP90 and SIBLING that consequently bind the active MMP2/9. TIMPs have an inhibitory effect on MMP2/9 activation. (**B**) CD10 metalloproteinase over-expression is correlated with cancer cell invasion in several tumors. Moreover, CD10 expression is up-regulated by transcription factor Twist1 which is considered a master inductor of EMT and thus, cancer cell invasion.

MMPs are a family of zinc-binding endopeptidases that participate in the ECM degradation molecular machinery during tumor invasion [137,138]. In fact, in order for tumor neo-vascularization and cell invasion processes to occur, degradation of the basement membrane as well as matrix remodeling are essential. Amongst the many known MMPs, MMP-2 and MMP-9 degrade gelatin as well as type IV collagen, the central component of the basement membrane. These MMPs are secreted in an inactive form and acquire their active form extracellularly [139,140]. Eustace *et al* in 2004, demonstrated that eHSP90 activates MMP-2 leading to increased tumor invasiveness in HT-1080 fibrosarcoma cells [141]. The association of eHSP90 with MMPs was further confirmed by Stellas *et al.* [142] in 2010 with the demonstration that eHSP90 participates in the activation of MMP-2 and MMP-9 in the process of breast cancer cell invasion (Figure 3A). Moreover fluorescence binding and affinity purification studies have shown that three members of the SIBLING family, BSP, DMP1 and OPN, activate pro-MMPs-, MMP-2 MMP-9 and MMP-3, respectively and subsequently bind to the catalytically active MMPs (Figure 3A). Additionally, SIBLING re-activate MMPs that have been de-activated by tissue or exogenous inhibitors [143]. ECM degradation and cancer cell invasion are inter-connected mechanisms that include basement membrane disintegration by actin-rich, finger-like cellular membrane projections located at the ventral side of the cell, named invadopodia [144]. MMP-2 and MMP-9 are enriched in the invadopodia where they contribute to ECM degradation *in vitro* and *in vivo* [144]. According to Jacob *et al.* [145], the intracellular transport and targeting of MMP-2 and MMP-9 to invadopodia during the breast cancer invasion process, is mediated by small monomeric Rab40b GTPase. More specifically, the transport of MMP-2 and MMP-9 from the trans-Golgi network (TGN) is mediated by secretory vesicles containing vesicle-associated membrane protein 4 (VAMP-4) and Rab40b (Figure 3A). Rab GTPases regulate various membrane transport steps including cargo sorting, vesicle budding, transport and targeting to the appropriate target compartment [146]. Jacob *et al.* showed that Rab40b knockdown, results in mis-targeting of MMP-2 and MMP-9 to lysosomes, where they are usually degraded. Overall, they identified Rab40b GTPase as the key regulator in MMP-2 and MMP-9 transport and targeting to the plasma membrane in the breast cancer invasion process. On the other hand, the down-regulation of MMP activity includes inactivation by extracellular tissue inhibitors of MMPs, named TIMPs [147] (Figure 3A).

Nowadays, it is established that the tumor invasion process is not only based on cancer cell migration but is also a result of the activity of normal cells [148]. In this context, it has been shown that tumor cells induce MMP expression and secretion by stromal cells, including fibroblasts, endothelial cells, and inflammatory cells via cell-cell contact or paracrine mechanisms. MMPs secreted by stromal cells, especiallyMMP-2, MMP-3 and MMP-9, contribute equally or even more to tumor cell invasion than MMPs secreted from cancer cells [137]. More specifically, Min *et al.* [137] demonstrated that poor overall survival is correlated with the expression of MMP-2 on stromal and tumor cells, as well as the expression of MMP-9 on tumor cells, and suggest that stromal MMP-2 may have a critical role in breast cancer aggressiveness. Finally MMPs promote neovascularization by inducing the secretion of heparin bound growth factors like VEGF and bFGF into their soluble pro-angiogenic forms. In this context, it has been reported that the production of the pro-angiogenic growth factor VEGF is induced by MMP-9 [149,150,151,152].

### 6.2. CD10

CD10, also called neprilysin and Common Acute Lymphoblastic Leukemia/Lymphoma Antigen (CALLA), belongs to the family of membrane bound, zinc-dependent metalloproteinases, members of which regulate the physiological action of various proteins by lowering their extracellular concentration available for receptor binding [153]. In fact, CD10 is involved in numerous biological activities through regulation of signal transduction of bioactive neuropeptides and vasoactive peptides [154,155]. In particular, it is expressed in the central nervous system, regulating various substrates such as encephalin, an opioid peptide liberated by neurons in response to pain, whilst polymorphisms in the CD10 gene augment the risk for Alzheimer’s disease. Moreover, CD10 is involved in regulating mechanisms of the immune system by controlling, through degradation, the activation of inflammatory peptides [156]. Additionally, it is considered as a stem cell regulator in breast, [157,158], lung [159], bone marrow [159] and adipose tissues [160]. The role of CD10 in tumor growth is still quite controversial. CD10 can be a good prognostic marker with various carcinomas such as cervical and non-small cell lung carcinomas [161,162] but on the other hand indicates poor prognosis with solid tumors such as gastric, pancreatic, and colorectal tumors where it is associated with disease progression and metastatic potential. Ikenaga *et al.* [163] reported that CD10^+^ pancreatic stellate cells promoted the invasiveness of pancreatic cancer cells *in vitro.* In particular, they suggested that CD10^+^ pancreatic stellate cells promoted invasiveness of tumor cells by secretion of MMP-3, and thus, ECM degradation. Moreover, increased expression of CD10 in tumor and stromal cells of bladder carcinoma is strongly correlated with tumor progression, invasion and metastasis in human bladder cancer [164,165]. Additionally, in invasive duct breast cancer, CD10 expression by stromal cells was positively correlated with a large tumor size, high tumor grade, presence of lymph node metastasis and low overall survival [166,167]. The molecular mechanisms underlying the role of CD10 in cancer invasion remain largely unclear. Nevertheless, very recently, Lee *et al.* associated CD10 over-expression in esophageal squamous cell carcinoma (ESCC) cells with activity of the transcriptional factor Twist 1. It had been already shown that Twist1 induces EMT in esophageal squamous cell carcinoma (ESCC) cell lines by up-regulating several genes [168]. Lee *et al.* showed that CD10 over-expression in ESCC cells is directly induced by Twist1, which binds to a specific site on the CD10 gene [169] (Figure 3B).

## 7. Chaperones

### 7.1. eHSP90

HSP90 is considered one of the most abundant cytoplasmic chaperones in unstressed normal cells, where it performs housekeeping functions, controlling the stability, activity, intracellular disposition and proteolytic turnover of a variety of client proteins. Moreover, HSP90 interacts with a great number of molecules that are involved in the development and/or survival of cancer cells, allowing mutant proteins to retain or gain function. Additionally, HSP90 allows tumor cells to tolerate genetic alterations, including mutations of critical signaling molecules that would otherwise be lethal. It actually functions as a biochemical buffer for the genetic instability found in cancers by stabilizing and permitting the accumulation of mutant proteins. As a result of this buffering capacity, phenotypic diversity within the tumor population increases and the evolution of invasive metastatic and drug resistant phenotype accelerates while permitting cancer cells to tolerate the imbalanced signaling that such oncoproteins create. HSP90 has also been identified in the extracellular milieu and has been shown to chaperone a finite number of extracellular proteins involved in cell migration and invasion [125].

While the existence of the cytoplasmic pool of HSP90 has been demonstrated and studied for the past three decades, it was not until 2004 that Sidera *et al.* identified an extracellular pool of HSP90 on the cell surface of developing neuronal cells that plays a critical role in cell motility. Through the development and use of a novel cell-impermeable, HSP90 function-blocking monoclonal antibody, namely mAb 4C5, they showed that eHSP90 is necessary for cell migration and is associated with actin-reorganization of migrating cells [170]. In the same year Eustace *et al.* revealed the presence of HSP90 on the cell surface and in the conditioned media of fibrosarcoma cells and associated the increased tumor invasiveness of these cells *in vitro* with the activation of MMP-2 by eHSP90 (Figure 3A) [141]. Furthermore, Becker *et al.* [171] showed the presence of eHSP90 on the surface of melanoma cells and correlated its over-expression with melanoma malignancy. In this context, Stellas *et al.* showed that anti-HSP90 mAb 4C5 inhibited cell invasion and metastasis in melanoma cells [172]. Additionally, an involvement of eHSP90 in the re-organization of the actin cytoskeleton and cancer cell invasion in prostate and bladder cancer was reported by Tsutsumi *et al.* [173], whilst Yang *et al.* demonstrated that hyperacetylation of eHSP90 promoted its extracellular location and caused increased breast cancer cell invasion [174]. Participation of eHSP90 in breast cancer cell invasion was also shown by Sidera *et al.* (2008) who revealed interaction of eHSP90 with the extracellular domain of HER-2 during the invasion process [125] (Figure 2B). It has been shown that several hostile environmental conditions such as serum starvation, hypoxia and oxidative stress trigger the extracellular localization of HSP90. In particular, according to Li *et al.*, hypoxic conditions trigger the accumulation of HIF-1, with the consequent secretion of HSP90 and the involvement of the latter in enhanced skin cell migration and wound healing. Hypoxia is a typical condition both in wound healing and in the tumor invasion processes. And, whilst normal cells release eHSP90 in response to tissue injury, this chaperone is constitutively secreted by tumors and promotes metastatic phenomena [175]. Finally, and given the previously mentioned capacity of CSC to survive under severe hypoxia conditions and nutrient shortage as well as to give rise to secondary tumors, our preliminary data suggest that eHSP90 is over-expressed on CSC [176], indicating a further involvement of this protein in cancer cell invasion and metastasis. Overall, increasing evidence indicates that eHSP90, either secreted in the ECM or loosely attached on the cell surface, acts as a chaperone for the activation of proteins involved in the processes of cancer cell invasion and metastasis and therefore might be of critical importance for the these processes.

### 7.2. eCdc37

Cdc37 was initially identified as part of a protein complex involving HSP90 and the Rous sarcoma virus-encoded oncogene pp60v-src. It interacts with various oncogenic protein kinases such as Raf1 and CDK4, the oncogenic mutant epidermal growth factor receptor tyrosine kinase EGFRvIII and the Peuth-Jeghers cancer syndrome-associated kinase [177]. Intracellularly, Cdc37 acts as a crucial co-chaperone in the HSP90 chaperone machinery, playing a decisive role in the maturation and/or stabilization of a large subset of protein kinases, implicated in signal transduction, proliferation and survival [178]. Cdc37 acts as an adaptor or scaffold, facilitating client kinase interaction with HSP90 [179] and subsequently by recruiting these client kinases into the HSP90 complex, it stabilizes and/or maintains them in a folding-competent conformation [180]. Many client proteins interact directly with both Cdc37 and HSP90 and their folding, maturation and stability depend on the activity of both chaperones. Vice versa, the complex relationship between Cdc37 and HSP90 is illustrated by the finding that their interaction is stabilized by the client protein [181]. In 2012, El Hamidieh *et al.* identified a cell surface pool of Cdc37 (eCdc37) that participated in breast cancer cell invasion. More specifically the authors showed that eCdc37 is localized on the surface of MDA-MB-453 and MDA-MB-231 breast cancer cells, where it is necessary for the motility of these cells and similarly to its intracellular counterpart it specifically interacts with eHSP90. Moreover, immunoprecipitation experiments using MDA-MB-453 and MDA-MB-231 cell lysates showed that eCdc37 also interacts with HER-2 and EGFR respectively. Thus, the authors concluded that eCdc37 possibly acts in a similar way to its intracellular counterpart, by functioning as a co-chaperone molecule for eHSP90 [182] (Figure 2B,C).

## 8. LRP-1

**Figure 4:**
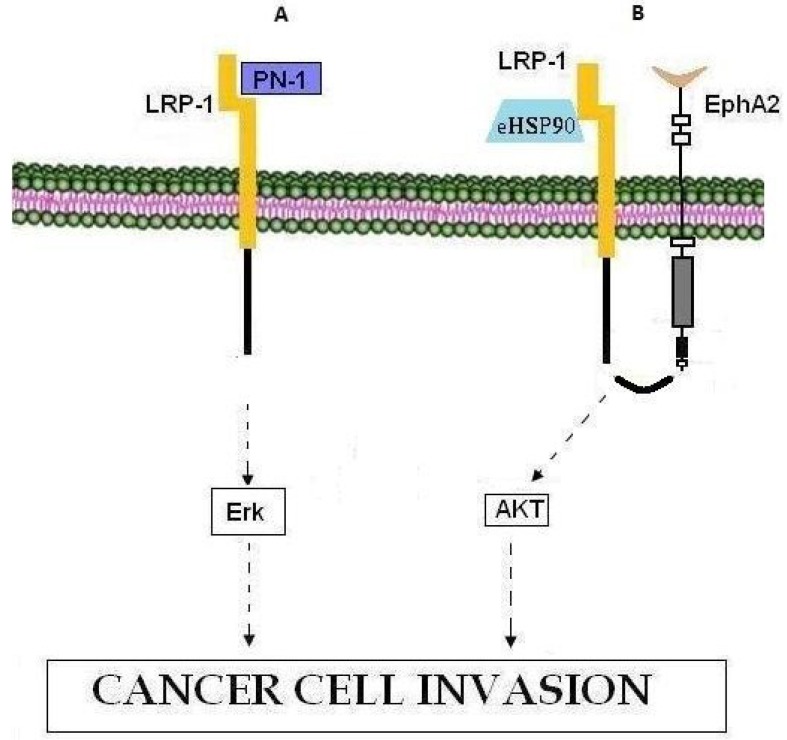
LRP1 involvement in cancer cell invasion. (**A**) PN-1 binding to LRP-1 leads to activation of the Erk signaling pathway and cell invasion. (**B**) The eHSP90-LRP1-EphA2 complex promotes tumor invasion through activation of the AKT signaling pathway.

LDL receptor-related protein-1 (LRP-1, also called CD91) is a member of the low-density lipoprotein (LDL) receptor superfamily, which includes at least 11 structurally related members. LRP-1 is a multifunctional molecule as it binds more than 40 ligands. Previous reports [175,183,184,185,186] have shown that nexin-1 (PN-1) binds LRP-1 and thus activates the ERK signaling pathway, controlling MMP-9 expression and inducing metastatic spread (Figure 4A) [183]. In this context, according to Montel *et al.* [187] silencing of LRP-1 in breast cancer has a negative effect on metastatic spread rather than on primary tumor growth. Moreover, it has been established that eHSP90 binds the extracellular sub-domain II of LRP-1 and activates downstream AKT1 and AKT2 kinase signaling pathways through the cytoplasmic NPVY motif of LRP-1, thus promoting cell motility and being essential for wound healing as well as tumor invasion [186]. In this context, during glioblastoma cell invasion, eHSP90-LRP1-mediated AKT signaling, necessitates the interaction of LRP-1 with pro-motility receptor tyrosine kinase EphA2 whose over-expression is common in cancers and is associated with oncogenic activity, cell invasiveness, metastatic potential and poor prognosis [188,189] (Figure 4B). Moreover, LRP-1 has been proposed to mediate the invasive properties of breast and thyroid cancer cells [184,187,190]. All in all, given its role in cancer cell invasiveness, LRP-1 represents a therapeutic target against metastasis whose inhibition will most probably constitute a selective pharmacological approach.

## 9. Conclusions

Cancer cell invasion represents the core of the complex phenomenon of metastasis and is the result of a dense and multifaceted network of molecular interactions taking place inside the cancer cell or within the cancer cell microenvironment. In the present review we have focused on the contribution of extracellular molecules in cancer cell invasion processes. Major components of the extracellular milieu include ECM proteins and their receptors, growth factors and their receptors as well as metalloproteinases. More recently, increasing evidence shows the participation of extracellular chaperone molecules such as eHSP90 and eCdc37 in cancer cell invasion. These chaperone proteins act either after secretion or as cell surface molecules loosely tethered to the cell membrane. All the above mentioned molecules participate in various aspects of the metastatic cascade such as ECM degradation, the stabilization and activation of extracellular oncogenic proteins, neo-vascularization and in cell-cell and cell-matrix interactions. In fact, they constitute the complex extracellular network of molecules that, in concert with their downstream intracellular signaling cascades, promote cancer cell invasion. Thus, one of the major tasks of oncology research is the development of inhibitors selectively targeting extracellular molecules that constitute a crucial inter-connection of multiple metastatic pathways.
